# Mechanisms of Globin Gene Regulation in Mammals

**DOI:** 10.1146/annurev-genet-020325-095743

**Published:** 2025-08-19

**Authors:** Ross C. Hardison

**Affiliations:** Department of Biochemistry and Molecular Biology, Center for Eukaryotic Gene Regulation, and Center for Computational Biology and Bioinformatics, The Pennsylvania State University, University Park, PA, USA

**Keywords:** regulatory elements, transcription factors, chromatin architecture, interaction frequencies, fetal hemoglobin reactivation

## Abstract

Studies of globin gene clusters have established many paradigms of gene regulation. This review focuses on the alpha- and beta-globin gene clusters of humans and mice, summarizing important insights from high-throughput biochemical assays and directed genetic dissections, emphasizing similarities across the types of gene clusters and between species. The overall arrangements and architectures are similar, with each gene cluster being localized within a topologically constrained unit of chromatin containing a multi-component enhancer (locus control region) and other regulatory elements bound by a similar set of transcription factors and co-activators. Differential expression of the globin genes within each cluster during ontogeny is associated with changes in contacts with the locus control region and involves the action of gene-specific repressors. Detailed study of the fetal beta-like *HBG1* and *HBG2* globin genes has revealed a remarkable diversity of regulatory pathways that provide candidates for therapeutic approaches to reactivate these genes for beta-hemoglobinopathies.

## 1. INTRODUCTION

Hemoglobin and the genes encoding it have intrigued generations of scientists, motivating intense research for almost two centuries. For example, studies in the 19^th^ century established the major physiological function of hemoglobin in gas transport via erythrocytes (18) and explored its biochemistry (66). The landmark determination of the three-dimensional structure of hemoglobin provided high resolution mechanistic insights into protein function (116).

Much of the keen interest in hemoglobin and its genes derives from their involvement in human diseases. Disorders of hemoglobin structure, such as in sickle cell disease, or inadequate hemoglobin production, such as in thalassemias, comprise the most common inherited diseases of humans (154). Haldane’s observation that these disorders are highly prevalent in equatorial regions across the world because carrier status confers resistance to malaria provided a powerful validation of Darwin’s theory of natural selection (57). Studies of these disorders led to many advances in our understanding of the molecular bases of disease. For instance, the discovery that an amino acid substitution in the beta-globin polypeptide caused sickle cell disease provided the first molecular definition of a mutation (72). Other variants in the globin genes also are associated with human pathologies, some resulting from changes in the protein sequence and others affecting the levels of globin protein (45; 52). The possibility that deeper understanding of molecular mechanisms of hemoglobin function and regulation of its genes would uncover novel avenues for therapies continues to be a major motivation for research into these issues. Indeed, recent progress led to the approval of two gene-based therapies for beta hemoglobinopathies in 2023 (3).

Many discoveries in biochemistry and genetics were made initially through studies of hemoglobin and its genes, facilitated by the high abundance of the protein and the mRNAs that encode it. The globin mRNAs were the first to be cloned as recombinant plasmids carrying copies of the mRNA (39), which were key reagents in the isolation of molecular clones containing the globin genes (49; 59; 81; 84). The availability of purified segments of genomic DNA containing genes of interest fueled a revolution in genetics, allowing the determination of DNA sequences, measurement of differential expression, discovery of genetic variants and regulatory elements, and investigation of the impacts of chromatin structure and epigenetic landscapes. Moreover, these molecular clones provided the starting point for intensive, directed mutagenesis followed by assessment of altered expression and function, i.e. reverse genetics approaches from genotype to phenotype. Technical advances have increased the impact of such studies. The development of high throughput genomic assays has expanded the scope of biochemical studies to cover entire genomes. Application of CRISPR based precision genome editing has enabled the study of directed mutations within more informative physiological and organismal contexts (31; 73; 136).

Studies of the cloned globin gene clusters have led to many advances in our understanding of gene regulation. This review summarizes some of the progress fueled by high throughput genomic assays and precision genome editing, including selected information on gene regulatory elements in the mammalian alpha- and beta-globin gene clusters, their spatial interactions, and the transcriptional regulatory proteins that bind to them, pointing out similarities among the gene clusters. Mechanistic models for gene regulation are summarized, with an emphasis on pathways regulating the perinatal silencing of fetal gamma-globin genes and how this can be reversed for the treatment of beta hemoglobinopathies. Much more information on these topics is available, and references are given to complementary reviews, along with a list of helpful resources.

## 2. THREE DIMENSIONS OF GLOBIN GENE REGULATION

Vertebrate hemoglobins are tetramers containing two alpha-like and two beta-like globin polypeptides, each bound by a heme. The genes encoding the alpha-like and beta-like globin polypeptides are organized into separate gene clusters on different chromosomes in reptiles, birds, and mammals (117). Expression of globin genes is regulated along three dimensions: tight cell type specificity, differential expression during development, and balance between the production of alpha-like and beta-like polypeptides.

Globin genes are expressed at extremely high levels during erythroid development, eventually making up the majority of mRNA and proteins produced (126). Small amounts of globins have been found in non-erythroid cells, including endothelial cells, in which they may play a role in regulating vascular tension (1). However, their high abundance expression is exclusive to erythroid cells.

**Figure 1:**
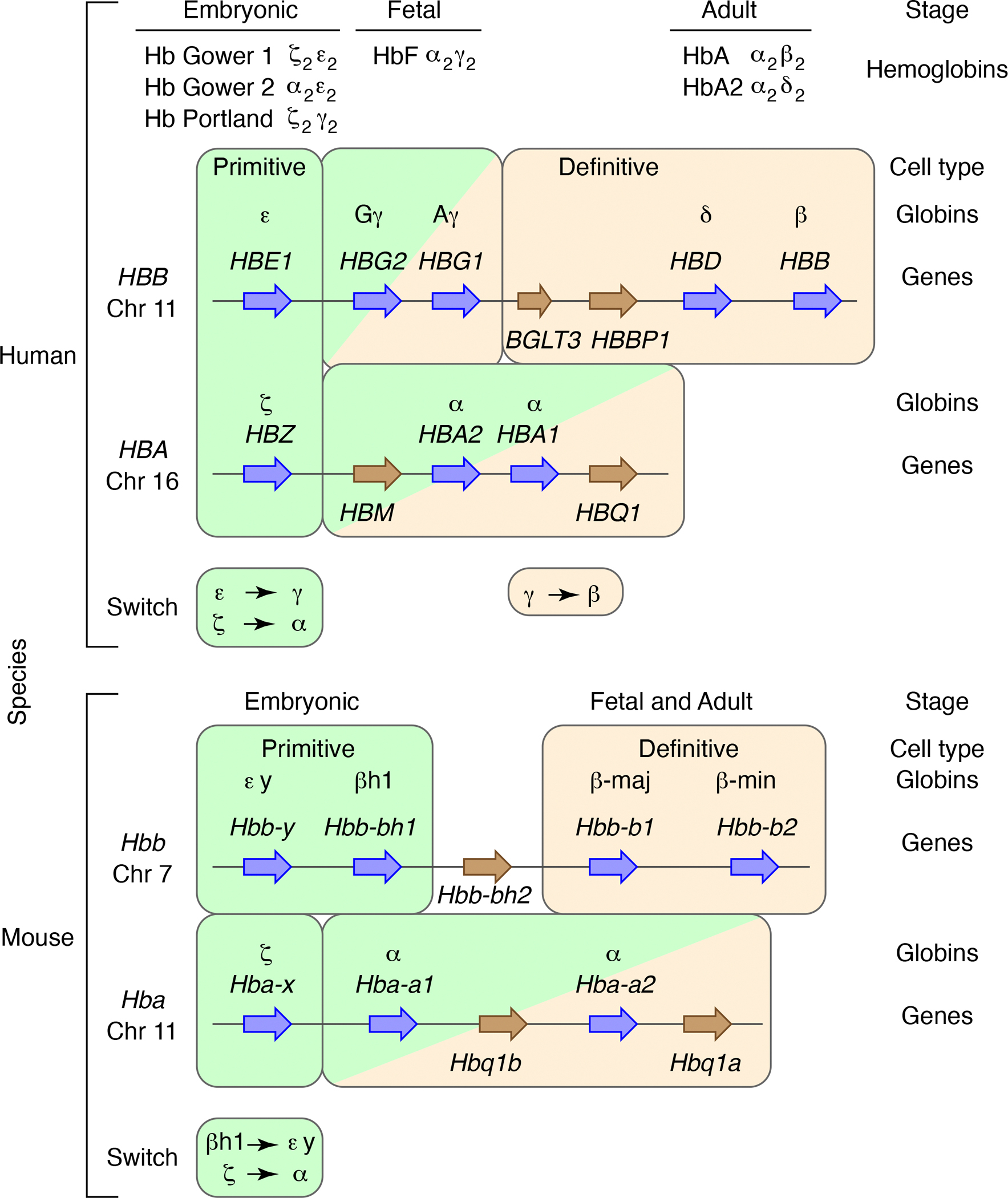
Differential expression of globin genes during development in humans and mice. The beta-like and alpha-like globin gene clusters are shown diagrammatically (not to scale) for human (top) and mouse (bottom), with blue arrows for genes that encode globin polypeptides that are components of hemoglobins and brown arrows for non-protein-coding genes, pseudogenes, and genes of unknown function. The rounded rectangles around sets of genes indicate their expression in the primitive (light green background) and definitive (tan background) cell lineages, with bicolor rectangles representing expression in both lineages. The developmental stages of expression are given, along with the hemoglobins produced in human erythroid cells.

Different hemoglobins are made at progressive stages of ontogeny in all vertebrates examined. In human embryos, the first wave of erythropoiesis, termed “primitive”, generates red blood cells containing three hemoglobin tetramers containing epsilon- or gamma-globin from the beta-like gene cluster and zeta- or alpha-globin from the alpha-like cluster (113) (Figure 1). Red blood cells from the second, “definitive” wave of erythropoiesis make hemoglobin F (HbF) during the fetal stage followed by replacement around birth predominantly by HbA. This fetal-to-adult hemoglobin switch within the definitive lineage occurs via a shift from expression of the *HBG1* and *HBG2* genes, encoding gamma-globins, in fetal cells to expression of the *HBB* and *HBD* genes, encoding beta- and delta-globin, respectively, in adult cells. The DNA sequences of the *HBG1* and *HBG2* genes are very similar. They are thought to have arisen from a gene duplication followed by rounds of gene conversion that keep some DNA segments almost identical, including the promoter regions (133). In this article, the term *HBG1,2* to refers to both genes. This switch from production of gamma-globins to beta-globin has been studied intensively because production of sufficient amounts of HbF in erythroid cells of patients with sickle cell anemia or beta-thalassemia can ameliorate many of the clinical symptoms of these diseases (15). The fetal specific expression of *HBG1,2* appears to have been acquired during the evolution of anthropoid primates, *i.e.*, humans, apes, and monkeys (58; 75). Notably, no comparable fetal-to-adult switch occurs in mouse erythroid cells; instead, the mouse *Hbb-b1* and *Hbb-b2* genes encoding beta-globin are expressed in both fetal and adult definitive erythroid cells (Figure 1). Production of embryonic alpha-like and beta-like globins switches in primitive erythrocytes. For the alpha-like globins of both species, production switches from zeta to alpha, and for the beta-like globins, the switch is from epsilon to gamma in humans and from betah1 to epsilon-y in mouse (113).

The third dimension of globin gene regulation is the balance between production of alpha-like and beta-like globins, which is essential for the formation of functional hemoglobin. An excess of either subunit type leads to nonfunctional protein aggregates, with pathological consequences for the erythroid cells. This imbalance drives the pathologies of thalassemias, which are inherited anemias that result from deficiency in the production of either alpha globins (alpha-thalassemias) or beta globins (beta-thalassemia) (129). Shared regulatory mechanisms between alpha-like and beta-like globin gene clusters likely contribute to their balanced, high-level production. Other activities reduce the negative impact of excess free globin chains, such as the protein chaperone alpha-hemoglobin stabilizing protein (157) and protein quality control mechanisms that degrade the unmatched globin chains (79; 142).

All three dimensions of regulation of globin genes share some common molecular features that are discussed in the next three sections: (a) expressed genes and their regulatory elements are in accessible chromatin with activity-associated histone modifications, (b) the regulatory elements are bound by transcription factors (TFs) that recruit co-factors, chromatin remodelers and/or RNA polymerase, and (c) distal regulatory elements are brought into close proximity to the promoters of target genes in three dimensional space by looping. These general mechanisms, largely established through early research on globin genes, are now known to regulate most or all vertebrate genes.

## 3. CHROMATIN ACCESSIBILITY IN GLOBIN GENE REGULATION

**Figure 2:**
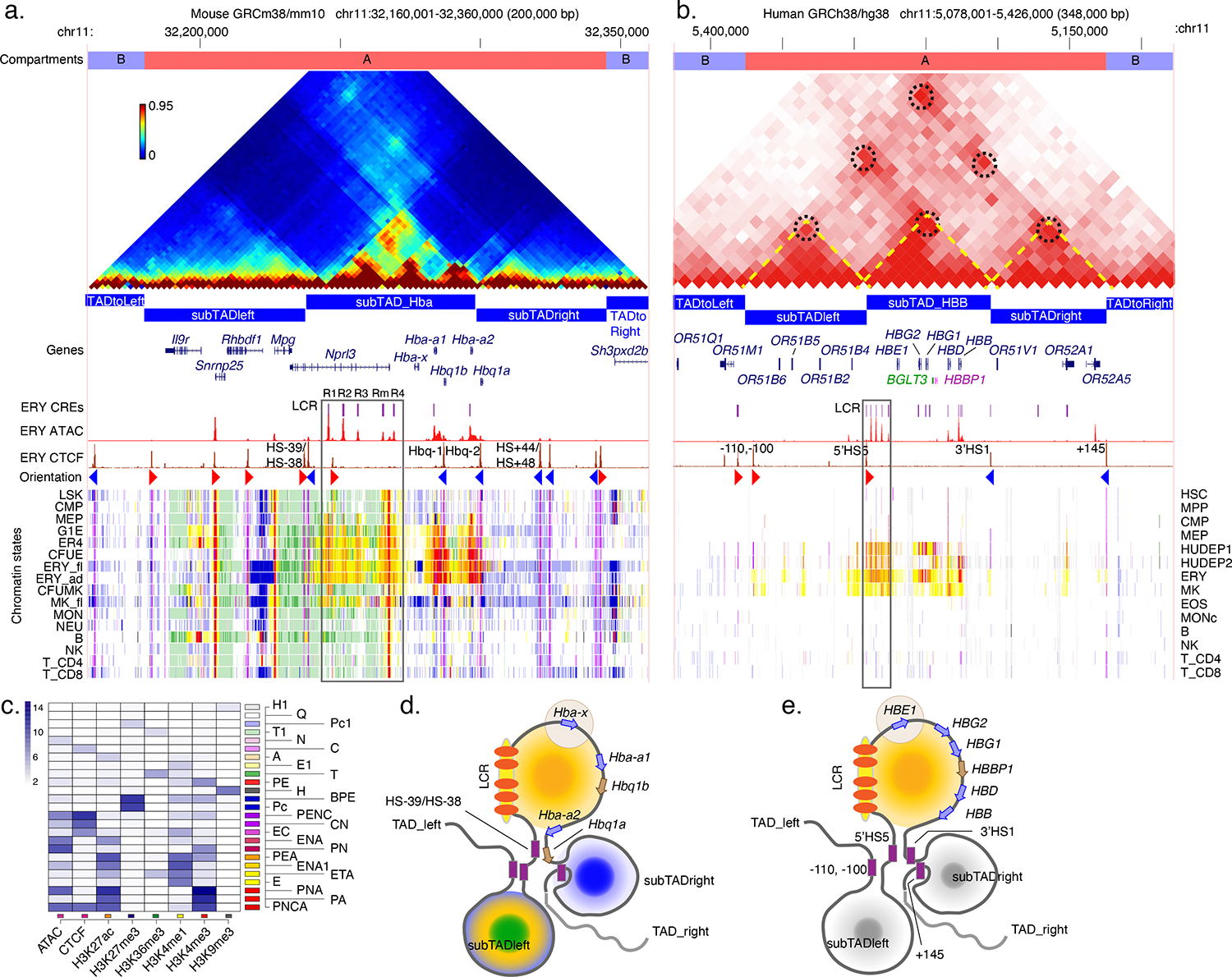
Chromatin architecture and epigenetic landscapes of the alpha- and beta-globin gene clusters. The compartments, topologically associated domains (TADs), genes, known *cis*-regulatory elements, chromatin accessibility (ATAC-seq) and CTCF occupancy in erythroblasts (fetal for mouse *Hba*, adult for human *HBB*), chromatin states across a range of hematopoietic stem, progenitor, and mature cell types for (*a*) the alpha-globin gene cluster in mouse and (*b*) the beta-globin gene cluster in humans. The chromosomal interval for each panel is centered on the subTAD encompassing the globin gene clusters and extends through the flanking subTADs to the beginnings of the adjacent TADs. The two-dimensional heatmaps convey the interaction frequencies between genomic intervals (fine divisions along the horizontal axis). The interaction frequencies for the mouse *Hba* gene locus (*a*) are from tiling 3C (110) shown on a multicolor heatmap scale. Panel image adapted from Reference (110) (CC BY license) with permission from the authors. The interaction frequencies for the *HBB* gene locus (*b*) are from Hi-C data (68) using a red (higher frequency) to white color scale. Panel image adapted from Reference (68) (CC BY NC 4.0 license) with permission from the authors. In (*b*), nodes of high interaction frequency characteristic of the anchors of chromatin loops are outlined with black dashed circles. The chromatin states were learned and assigned jointly on epigenetic data (chromatin accessibility, histone modifications, and CTCF occupancy) from both human and mouse hematopoietic cells (164). (*c*) The contribution of each of eight epigenetic features (columns) to each of 25 chromatin states (rows) is indicated on a white to blue scale, along with colors and labels for each state. Abbreviations for labeling the chromatin states are P = Promoter like, E = Enhancer like, N = Nuclease accessible, H = Heterochromatin, Q = Quiescent, A = Active, C = CTCF, B = Bivalent, T = Transcribed. Diagrammatic interpretations of the architecture and epigenetic landscapes are shown for (*d*) the mouse *Hba* cluster and (*e*) the human *HBB* cluster, with loops for each subTAD emanating from a cluster of anchors (not all are known). The subTADs are colored by the predominant chromatin states in fetal and adult erythroblasts, with an LCR (red ovals for each HS) and the globin genes in the middle subTAD. The light brown disk around the embryonic genes indicates a repressed zone within an otherwise active subTAD.

**Figure 3:**
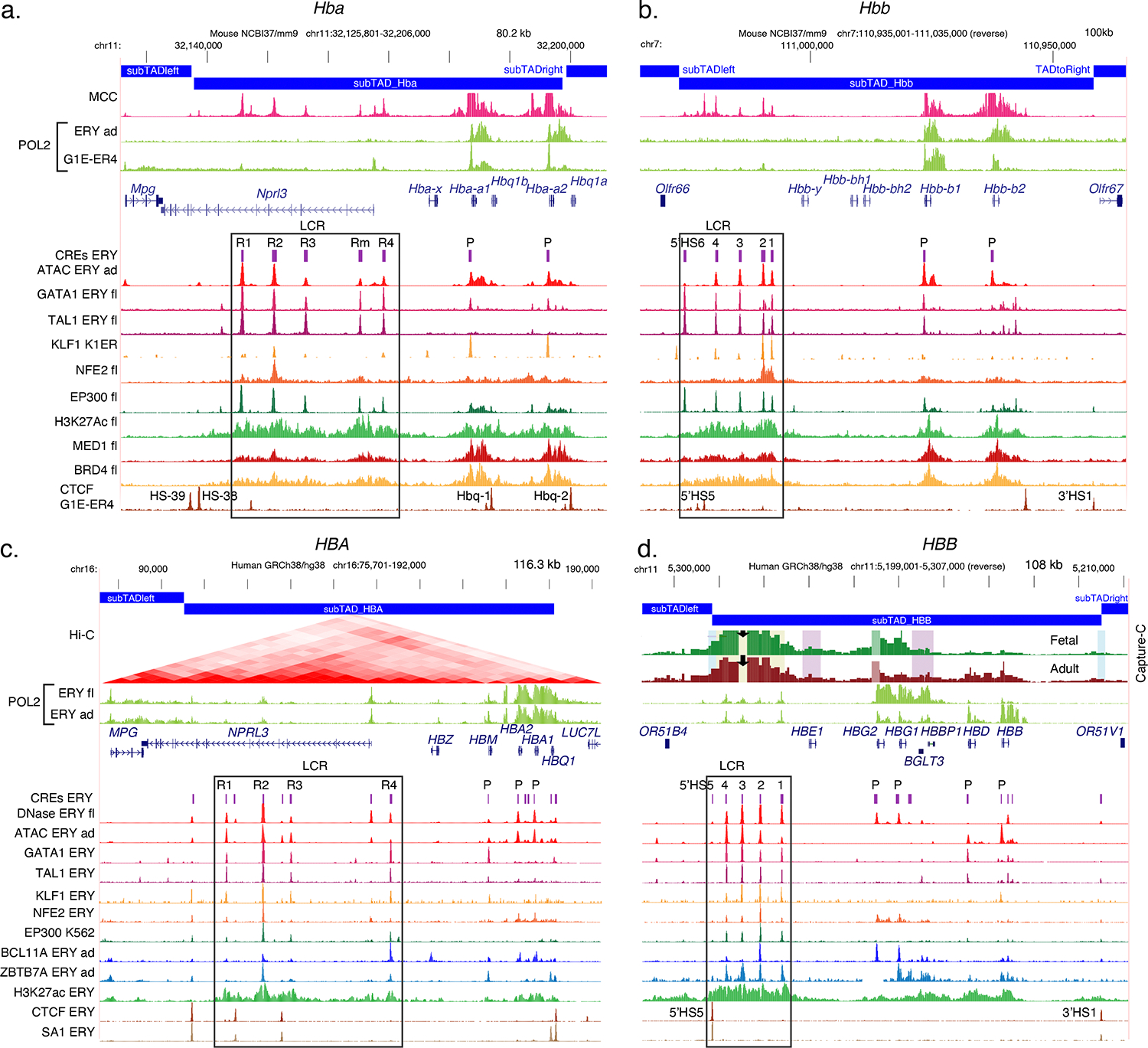
Gene regulatory elements and interaction frequencies in globin gene clusters. For the alpha-globin gene clusters (*a* and *c*) and beta-globin gene clusters (*b* and *d*) of mice (*a* and *b*) and humans (*c* and *d*), the genes, known CREs, and chromatin accessibility profiles are shown, along with signal tracks for chromatin interaction frequencies, occupancy by POL2, GATA1, TAL1, KLF1, NFE2, EP300, CTCF, and acetylation of H3K27. Signal tracks for Mediator (MED1) and BRD4 are shown for mouse, and BCL11A, ZBTB7A, and the cohesin subunit SA1 for human. The interaction frequencies are from different methodologies from Hi-C (human *HBA* cluster, *c*) to higher resolution Capture-C (human *HBB*, *d*) to base-pair resolution with Micro-Capture-C (MCC in mouse, *a* and *b*). References for data in the signal tracks are in a Supplementary Table. Panel *d* image for Capture-C adapted from Reference (68) (CC BY NC 4.0 license) with permission from the authors.

Chromatin accessibility is a major feature of the mechanisms that determine cell type specificity and developmental control of expression because the expressed globin genes are in active, accessible chromatin specifically in erythroid cells. Early studies revealed that active genes are located in euchromatin (89), and a landmark study by Weintraub and Groudine (155) showed that specific genes, such as globin genes, are in broad regions of open, nuclease-accessible chromatin when active but in closed chromatin when inactive. Subsequent studies over 50 years have greatly expanded the scope and refined the resolution of this strong connection between gene expression and chromatin accessibility (e.g. 103). Furthermore, within the broadly nuclease-accessible regions, specific sites are hypersensitive to cleavage by nucleases or transposases. These hypersensitive sites (HSs) mark regulatory elements bound by specific TFs and chromatin remodelers (e.g. 151). The HSs determined by the efficient and robust method, assay for transposase-accessible chromatin with sequencing (ATAC-seq) (23), are shown for globin gene clusters in Figures 2 and 3. The HSs tend to be flanked by chromatin with histone modifications characteristic of gene activation, such as H3K27ac, and more specifically histone H3K4me3 at active promoters and H3K3me1 around active enhancers (61).

## 4. TRANSCRIPTION FACTORS AND COMPLEXES IN GLOBIN GENE REGULATION

Specific binding of lineage-specific and generalized TFs to regulatory elements is a major component of all three dimensions of regulation. Unique combinatorial interactions between these TFs and their *cis*-acting DNA elements determine the cell type specificity of gene expression. These TF combinations play similar roles in activating or repressing genes at progressive stages of development. Likewise, they play similar roles for both alpha-like and beta-like globin genes. Erythroid-specific TFs regulate not only globin genes, but also most other genes expressed specifically in the erythroid lineage (141; 158). Examples of binding profiles for many of these regulators, determined by chromatin immunoprecipitation analyzed by sequencing (ChIP-seq) and cleavage under target and release using nuclease (CUT&RUN), are shown in Figure 3.

The major lineage-restricted TFs include GATA1, TAL1, NFE2, and KLF1 (44). Gene knockout experiments show that these TFs are needed for erythroid differentiation, albeit at different stages of development (108). GATA1 is a zinc-finger TF that was the first discovered member of a small class of TFs that share the core binding site motif GATA (40; 43; 146). One paralog, GATA2, is an important regulator in hematopoietic stem and progenitor cells (HSPCs). TAL1 is a basic helix-loop-helix (bHLH) TF that acts as a heterodimer with other bHLH factors such as E47 (120). GATA1 and TAL1 bind together along with the linker protein LMO2 and the looping factor LDB1 (2; 153) at many regulatory elements of genes activated during erythroid maturation (145; 163). Several of these proteins are also part of a larger heptad complex, which includes GATA2, TAL1, LMO2, FLI1, ERG, LYL1, and RUNX1, that regulates many genes in HSPCs and other myeloid lineages and may prime CREs for activity during lineage-specific maturation (139). The GATA1 protein also binds FOG1 (ZFPM1) as a cofactor (147). At many regulatory sites, binding switches from GATA2 to GATA1 during differentiation (38). This GATA switch can lead to gene repression, such as at the *Gata2* (54) and *Kit* loci (74), but it also correlates with erythroid gene activation at many loci (162).

The zinc finger protein KLF1 was identified initially as a TF acting at the beta-globin gene promoter (104), and it is now known to many erythroid genes (115) by binding at promoters (141) and distal elements (106). NFE2 (nuclear factor erythroid 2) is a member of the AP1 family of leucine zipper TFs. It is a heterodimer of p45 and a MAF protein such as MAFK, and it binds in the strongest enhancers in the globin gene clusters to increase expression (9; 128).

TFs bound to regulatory elements can recruit enzyme complexes that remodel and modify chromatin to enable gene expression. Prominent among these complexes are the co-activator and histone acetyl transferase EP300 (21), the multi-subunit chromatin remodeling complex BAF (11), the mediator complex that bridges between gene-specific TFs and the transcriptional machinery (138), and BRD4, a multifunctional chromatin “reader” that binds to acetylated histones (137). The GATA1 cofactor FOG1 and other TFs can recruit the nucleosome remodeling and deacetylase (NuRD) complex for repression (65).

While it is common to discuss the impact of TFs as binary, i.e. functioning when present and not when absent, the regulatory effects of TFs are concentration dependent, and quantitative changes in TF protein levels can be key determinants of cell fate decisions during erythropoiesis and other processes. Applying targeted mass spectrometry to measure the abundance of about 100 TFs, co-factors, and components of the transcription machinery during human erythropoiesis has led to important insights (53). These experiments show that the protein abundance of many TFs is not reflected in the mRNA abundance, emphasizing the need for quantitative protein measurements. Strikingly, co-repressors are in large excess over co-activators in the nucleus, with TFs falling in an intermediate range of abundance. Thus, the overall nuclear environment is largely repressive, indicating that TFs compete for limited numbers of co-activator molecules within a repressive milieu. This result supports the hypothesis that repression can result from losing in a competition with other genes for co-activators (27).

## 5. CHROMATIN ARCHITECTURE: INTERACTIONS AMONG REGULATORY ELEMENTS

Gene activity has long been associated with location in the nucleus. Transcribed genes are located in euchromatin away from the nuclear periphery (89), and specific loci, including beta-globin genes, move during activation to regions of high transcriptional activity (109; 130) between the territories occupied by each chromosome (19).

An orthogonal approach to examine chromatin architecture at a higher resolution is the estimation of interaction frequencies among segments of genomic DNA using chromosome conformation capture (3C) techniques (35). These methods rely on fixing the structure in chromatin or nuclei *in situ*, followed by DNA cleavage, ligation of proximal segments, and sequencing to reveal junctions. The frequency of novel junctions provides an estimate of the contact frequency between segments of genomic DNA. Advances in 3-D proximity detection techniques have increased their scope and resolution (4). Statistical and computational analyses of the contact frequencies have revealed insights into chromatin architecture at multiple layers. Some of these results are illustrated for the alpha-globin gene cluster in mouse (110) and the beta-globin gene cluster in humans (26; 68; 93; 134; 143) in Figures 2 and 3.

A correlation analysis of the genome-wide interaction frequencies from Hi-C reveals two types of large, megabase sized compartments, in each of which the DNA tends to associate more with other DNA in the same compartment (88). The A and B compartments correspond to active euchromatin and inactive heterochromatin, respectively. Both types of globin gene clusters are located in A compartments in erythroid cells (Figure 2). A higher resolution analysis revealed regions of genomic DNA, termed topologically associating domains or TADs. Sequences within a TAD interact more frequently with each other than with sequences in other TADs. The TADs themselves are hierarchically organized into smaller domains of high interactions, called subTADs. For both globin gene clusters, the genes and major distal regulatory elements reside within an internal subTAD (Figure 2). Many TAD and subTAD boundaries are occupied by the structural DNA-binding protein CTCF (CTCC-binding factor), with the CTCF proteins at the two ends of the TADs bound in opposing orientations. A proposed mechanism for establishing the boundaries of TADs and subTADs is the extrusion of chromatin by cohesin to form chromatin loops, with processive extrusion limited by encountering CTCF molecules bound in opposite orientations (50). This proposed role of CTCF-bound sites as boundary elements was examined in the mouse alpha-globin gene cluster by an extensive deletional analysis, which showed that CTCF-bound sites at HS-38 and HS-39 kb are required to form the left border of the *Hba* subTAD. However, the right border of the *Hba* subTAD is not dependent on the CTCF-bound site in the vicinity, Hbq-2, but rather it appears that the actively transcribed *Hba2* gene itself acts as the right boundary (32; 76) (Figure 2A). Thus, other elements in addition to bound CTCF can be used to establish the boundaries of TADs and subTADs.

The subTADs containing the mouse *Hba* gene cluster and the human *HBB* gene cluster also encompass their major proximal and distal regulatory regions (see Section 6). The schematic illustrations (Figure 2, bottom panels) show only major loops encompassing each subTAD, but higher resolution data reveal many chromatin interactions within each subTAD that correlate with activation of specific genes (Figure 2A, note the three triangles of higher interactions within the *Hba* subTAD; Section 7). The region of general DNase sensitivity in erythroid cells identified in early studies (156) has been mapped in detail for the mouse beta-globin gene cluster (24). It is largely homologous to the subTAD containing the human *HBB* cluster, suggesting that the region of general chromatin accessibility may correspond to a subTAD. This possibility would provide an architectural explanation, i.e. a subTAD, for the general accessibility, which is conceptually similar to the early proposals of a chromatin loop in the accessible chromatin (28).

Covalent modifications of histone tails are associated with major processes in gene expression and regulation (61; 62). The locations of chromatin with specific histone modifications have been mapped genome-wide by ChIP-seq in different hematopoietic cell types, including HSPCs, lineage-committed progenitors, and maturing cells. An integrative analysis of these data uses machine learning to reveal the most common co-occurring histone modifications as chromatin states and to assign each DNA segment across a genome to a chromatin state. For visualization, each state has a color (Figure 2c), e.g., with shades of red and yellow for states associated with gene activation, green for transcriptional elongation, blue for the repressive polycomb mark H3K27me3, and white for a quiescent state with no detectable epigenetic features, thereby displaying epigenetic landscapes that reveal evidence of major processes in expression and regulation across multiple cell types (164; 165) (Figure 2a and b). In both the alpha- and beta-globin gene clusters, the genes and their distal regulatory elements are in states indicating active transcription in erythroid cells and in the population of megakaryocyte-erythroid progenitor (MEP) cells, but not in the non-erythroid hematopoietic cell types, consistent with their expression patterns. By contrast, the epigenetic landscape differs between the two types of globin gene clusters for other, non-globin genes. Several genes around the alpha-globin genes are widely expressed across blood cell types, including *Nprl3*, which contains distal regulatory elements of alpha-like globin genes, along with *Mpg* and *Snrnp25* in the left subTAD. These non-globin genes present a pattern of active promoter (red) and transcriptional elongation (green) states in all the hematopoietic cell types. The beta-globin gene cluster is flanked by olfactory receptor (OR) genes, which are expressed only in nasal epithelial cells; they are mainly in a quiescent state in all blood cells. Furthermore, the chromatin states in non-erythroid cells differ for the two types of globin gene clusters, with evidence of polycomb repressed (blue) states for the *Hba* gene cluster but quiescent (white) states for the *HBB* gene cluster, which suggests that their silencing occurs by different mechanisms. While the basis for these differences needs direct experimental investigation, one hypothesis to consider is that the activation state of the surrounding genes in non-erythroid cells could be a factor. For example, since the alpha-globin gene clusters are embedded in close proximity with widely-expressed genes, they are surrounded by active chromatin in non-erythroid cells, and thus, they may require an active silencing mechanism, such as the continuous deposition of the polycomb modification H3K27me3. In contrast, the beta-globin gene clusters are surrounded by olfactory receptor genes that are not expressed in any blood cell types, and a more passive silencing mechanism, such as sequestering the genes in quiescent heterochromatin, could spread to encompass the beta-globin genes in non-erythroid cell types.

Chromatin architecture plays important but distinct roles in the three dimensions of regulation. For cell type specificity, the architecture discussed here for erythroid cells is not observed in non-erythroid cell types, especially for the local interactions (110; 112). For differential expression, similar subTAD structures around the *HBB* cluster are observed in both fetal and adult erythroid cells, but different local interactions of genes with the distal regulator are revealed at higher resolution (68) (Section 7). The alpha-like and beta-like globin gene clusters are both in subTADs with a powerful enhancer, enabling high level expression of the genes, which can contribute to balanced production of globins.

## 6. GENE REGULATORY ELEMENTS IN ALPHA- AND BETA-GLOBIN GENE CLUSTERS

Multiple regulatory elements that act in *cis* to the globin genes, *cis*-regulatory elements or CREs, are distributed throughout the alpha- and beta-globin gene clusters of both mouse and humans (Figure 3, CREs ERY). The general arrangement of CREs is similar across the four gene clusters, with a promoter (P) at the 5’ end of each active gene, a distal control region marked by multiple HSs [a locus control region (LCR) or super enhancer], and some additional enhancers within or in the 3’ flank of the genes.

### 6.1. Promoters

The promoters of globin genes include the core promoter and the proximal promoter. The core promoter is the DNA segment on which RNA polymerase II (POL2) and the rest of the preinitiation complex assembles around the transcription start site (TSS) (100; 112). All globin gene core promoters contain a TATA motif, which is the binding site for TBP, along with nearby sites for binding general TFs. The proximal promoter upstream from the core promoter contains binding sites for activators and repressors that regulate the frequency of initiation from the core promoter (100). Most if not all proximal promoters of globin genes contain a CCAAT box, the binding site for NF-Y and other proteins, and a binding site for KLF1 or a related family member. While NF-Y is a generally expressed TF, KLF1 is largely erythroid specific. Binding of KLF1 to the active genes has been demonstrated by ChIP-seq (106; 141) (Figure 3, KLF1 tracks).

For many genes, enhancers encode the developmental regulatory information, but in contrast, many of the elements for developmental regulation of the globin genes reside in the promoters. This is particularly well established for the intensively studied promoters of the *HBG1*,*2* genes, which are regulated by stage-specific repressors, as discussed in Section 8.

Promoter activity is reflected by RNA POL2 occupancy (Figure 3). ChIP-seq data show that POL2 covers the active globin genes starting at the promoters and extending downstream from the polyA addition site to produce nascent, unprocessed transcripts. Thus, transcription units are considerably longer than the annotated globin genes due to the continuation of nascent transcripts past the polyA addition site (64; 123). The pattern of coverage by POL2 fits with the differential expression of *HBG1,2* and *HBB* genes in human fetal and adult erythroblasts, respectively (Figure 3D).

The promoters for the alpha-like globin genes of mammals are in CpG islands. The promoters for most human and mouse genes are in CpG islands, where their distinctive sequences and lack of DNA methylation are important features in their activity (86). Promoter CpG islands of many genes lack TATA and CCAAT motifs, but those of the alpha-globin genes have these motifs within their CpG-rich contexts.

While the most abundant transcription from the globin gene promoters produces precursor to mRNA, sensitive assays of nascent transcripts reveals additional transcription of many genes on the opposing strand proceeding “upstream” (12), similar to the bidirectional transcription from many enhancers (8). These upstream antisense transcripts are of considerable interest because of potential roles in regulating expression of the coding genes (12; 94).

### 6.2. Locus control regions

A major distal regulatory region is located at the 5’ (left) end of the alpha- and beta-globin gene clusters in both species (Figure 3). The distal regulatory regions are large (about 25 kb, ranging from 16.5 kb to 38.7 kb for the human *HBB* and *HBA* gene clusters, respectively) and marked by several HSs indicative of multiple regulatory elements within them. Furthermore, chromatin containing these distal regulatory regions is highly acetylated at histone H3K27 and is bound by the mediator component MED1 and by BRD4 (20; 60) (Figure 3). Thus, the distal regulators of both types of gene clusters fulfill the criteria for super enhancers (159), and that is the name used most often for this control region in the alpha-globin gene clusters (20; 60). However, the distal regulators in both types of clusters share properties that inspired the name locus control region (LCR) for this regulatory region in the beta-globin gene cluster. In this review, the term LCR is used for the multi-component distal regulatory region for both the alpha- and beta-globin gene clusters.

The LCRs are strong enhancers that facilitate high level expression of any of the genes in the globin loci in erythroid cells. The LCR for the *HBB* cluster was discovered initially as a set of distal DNase HSs (47; 56; 148). The requirement of the LCR for expression was deduced from observations in human genetics and extensive mutational analysis in model systems. Some thalassemias result from deletions that remove critical parts of the LCR, both for the beta-like (46) and alpha-like (63) globin gene clusters, resulting in low expression of the target genes. In transgenic mouse experiments, the globin genes in large clones of recombinant DNA containing the beta-like globin gene cluster are expressed at high levels only when the LCR or key components of it are present (56). Similarly, the LCR strongly enhances expression of globin genes in transfected cells (e.g., 105; 149). Specific individual HSs within the LCRs have significant enhancer activity even when separated from the rest of the LCR, especially 5’HS2 (124; 140) and 5’HS3 (118) in the beta-like globin gene cluster and R2 in the alpha-like globin gene cluster (60; 132). Much of the enhancement activity in transfected cells maps to binding sites for the TF NFE2 (107) (Figure 3).

The *HBB* cluster LCR can also protect transgenes in mice from position effects, *i.e.*, the repressive effect of many chromosomal sites of integration. In early studies, this protection from position effects was considered a defining property of the LCR (56). This protection could be explained at least in part by the presence of an insulator at the 5’ end of the LCR. Insulators can block the activating effect of an enhancer on a target promoter when placed between the enhancer and promoter. A strong insulator is located in a regulatory element 5’ to the chicken beta-globin gene cluster, 5’HS4 (29), and its insulation activity is due in part to the binding of the structural protein CTCF (16). The chicken 5’ HS4 site may be analogous to the CTCF-bound 5’ HS5 sites in human and mouse *HBB* LCRs (41). However, the insulator activities of the latter sites are not as strong as that of chicken 5’HS4. The 5’HS5 sites in the LCRs are close to the boundaries of the subTADs containing the beta-like globin gene clusters, suggesting that they are involved in a related role, that of boundary formation, similar to the role of the LCR-proximal CTCF sites, HS-38 and HS-39, in boundary formation in the alpha-like globin gene cluster. The ability of the LCR to prevent position effects on transgenes may be a consequence of the natural role of 5’HS5 as a boundary element. This ability to block some position effects was important in the development of vectors used for gene therapy, and both insulating and enhancing elements from the LCRs have been incorporated into vectors used for expression in erythroid cells (25).

The pattern of TF binding at each of the HSs of the LCR is remarkably similar for both the alpha-globin and beta-globin LCRs in mouse and human (summarized in Figure 3). The major erythroid TFs GATA1 and TAL1 bind to all LCR HSs except those bound by CTCF. The co-activator and histone acetyl transferase, EP300, also occupies these HSs, perhaps reflecting its recruitment by the GATA1-TAL1 complex. Another erythroid master regulator, KLF1, and the activator NFE2 are bound to several of the HSs in the LCR, including those with independent enhancer activity. RNA POL2 is also present at low levels at the HSs of the LCRs of the alpha- and beta-globin gene clusters, producing RNAs that are associated with enhancer activity (82).

The HSs of the LCR interact extensively with each other and with target globin genes as revealed in 3C-type chromatin interaction measurements. These interactions are apparent in the all-against-all Hi-C data (88; 122) (Figure 3c), in higher resolution Capture-C data (Figure 3d), which shows interactions across the locus with a specified anchor (34), and in Micro-Capture-C (MCC) data (Figure 3a and b), which measures all interactions with the viewpoint (the promoters of the *Hba-a1* and *Hba-a2* genes in this case) across the locus at base-pair resolution (67). The interactions of the LCR HSs with globin genes fits with a looping model for bringing the distal enhancers in proximity with the target promoters (51). The interaction frequencies change during the developmental switch in the human *HBB* cluster, with higher interactions of the LCR with the *HBG1,2* genes in fetal erythroblasts and a shift to higher interactions with the *HBD* and *HBB* genes in adult erythroblasts (13; 68; 93) (Figure 3d). The region containing the non-coding RNA gene *BGLT3* and the nearby pseudogene *HBBP1* participates in this switch, in part by regulating contact with the LCR (68).

Several lines of evidence suggest that the LCR HSs are working together. The underlying DNA segments in the HSs are bound by many of the same TFs, they are covered by a contiguous zone of acetylated chromatin with co-activators bound, and they contact each other and the globin genes. The LCR has been proposed to form a chromatin hub with interactions among the HSs (112; 114). One prediction of a proposed LCR structure with interacting elements working together is that these elements should display synergism, specifically, the enhancement by the combined HSs should be greater than the sum of the activities of individual elements. This prediction has been rigorously tested by directed mutation of the LCR within the chromosomes in mice. In contrast to the predicted synergy, when single and multiple elements were deleted from the LCR of either the beta-like (17) or the alpha-like (60) globin gene clusters, only additive effects on globin gene expression were observed. Thus, these genetic analyses *in vivo* do not support models that predict synergy among the elements of the LCRs, which raises a conundrum in interpreting the extensive similarity in biochemical features for these elements.

Further dissection of LCR function revealed two distinct categories of distal elements in the LCR: classical enhancers and facilitators. The deletion of some individual HSs within the LCR caused a significant reduction in expression of the target globin genes; specifically, these are 5’HS3 and 5’HS2 in the beta-like gene cluster LCR (17) and R1 and R2 in the alpha-like gene cluster LCR (60). These elements also function as enhancers in a range of assays, and hence they are considered classical enhancers. Remarkably, synthetic alleles containing only one classical enhancer HS gave much less expression that expected based on the phenotypes of the deletion alleles, indicating that other regions within the LCR were needed for high level expression. Building additional synthetic alleles with informative combinations of HSs showed that HSs that lack enhancer activity do play a role in increasing the transcriptional stimulation by the classical enhancer (20). These LCR elements, specifically R3, Rm, and R4 in the alpha-like gene cluster, are facilitators. They present the biochemical hallmarks of enhancers, and while they have little or no enhancer activity on their own, they function to modulate the activity of classical enhancer elements. The ability of a facilitator to boost the effects of classical enhancers depends on its position relative to the target gene, with stronger activity observed at more gene-proximal positions. Facilitators are not limited to the *Hba* cluster LCR; the 5’ HS1 element from the *Hbb* cluster LCR also acts as a facilitator (20). These studies reveal at least two distinctly different functions for elements within the LCRs, despite the striking similarities in their biochemical signatures.

It is important to keep in mind that the biochemical data (from ChIP-seq, ATAC-seq, etc.) and interaction frequency measurements provide static views of chromatin in the cells assayed. In reality, chromatin interactions are likely dynamic, and the elements of the LCR may not be interacting with each other or the target genes at the same time. Interactions of individual LCR elements in a series rather than simultaneously would fit with additive effects of the elements in LCR function (6; 111). Further studies with orthogonal methods, such as visualization of the dynamic behavior of single chromosomes in cells by super-resolution microscopy, are needed to understand more completely the roles of the LCR and its elements.

The consistent location of the LCR at the 5’ ends of both globin gene clusters raised the possibility that the LCR acts directionally, only affecting genes downstream of it, and possibly having differing effects on the target genes depending on their distance from the LCR. While independence of orientation has been considered a defining property of individual enhancers, it is possible that groups of enhancers and facilitators in an LCR or super enhancer might show a polarity their activity. Early experiments to test this hypothesis utilized mice carrying long clones of human DNA encompassing the *HBB* LCR and globin genes integrated randomly in the mouse genome. Flipping the orientation of the LCR caused a substantial reduction in expression of the *HBB* gene in adult erythroid cells, indicating that the enhancement activity from the LCR did not extend in the reverse direction (20). Recent experiments used genome editing to reverse the orientation of the LCR in the native alpha-globin gene cluster in mice. Flipping of the LCR substantially reduced expression of the *Hba* genes in erythroid cells while also greatly increasing expression of the upstream genes *Snrnp25* and *Rhbdf1* (76; 77). These results show a clear polarity in the effect of the LCR. Since a dependence on orientation is not considered to be a feature of classical enhancers, it appears that orientation dependent, polar effects of grouped enhancers and facilitators within an LCR or super enhancer represent an emergent property, providing further support for cooperation among individual elements. While more studies are needed to elucidate the mechanisms for the orientation-dependent effects of the LCR, the positional dependence of the effect of facilitators suggests that they may provide a directionality to the activity of the LCR (20; 77).

### 6.3. Distal CREs in and around globin genes

CREs have been mapped within and close to the globin genes. Early studies identified an enhancer within a human *HBB* intron and 3’ to the gene (10), and an enhancer was mapped 3’ to the human *HBG1* gene (22) (CREs ERY track of Figure 3d). Studies of alpha-globin genes indicated that much of their CpG island, including sequences within the genes, is needed for transcriptional activity (135). Current maps of chromatin accessibility and TF occupancy support the presence of gene-internal and nearby candidate CREs (Figure 3), but the mechanisms by which they regulate target genes are not clear. Thus, additional elements within and close to the genes may be playing roles that could be productive subjects for future research.

## 7. REGULATION BY SHIFTS IN INTERACTIONS OF GENES WITH THE LCR

**Figure 4:**
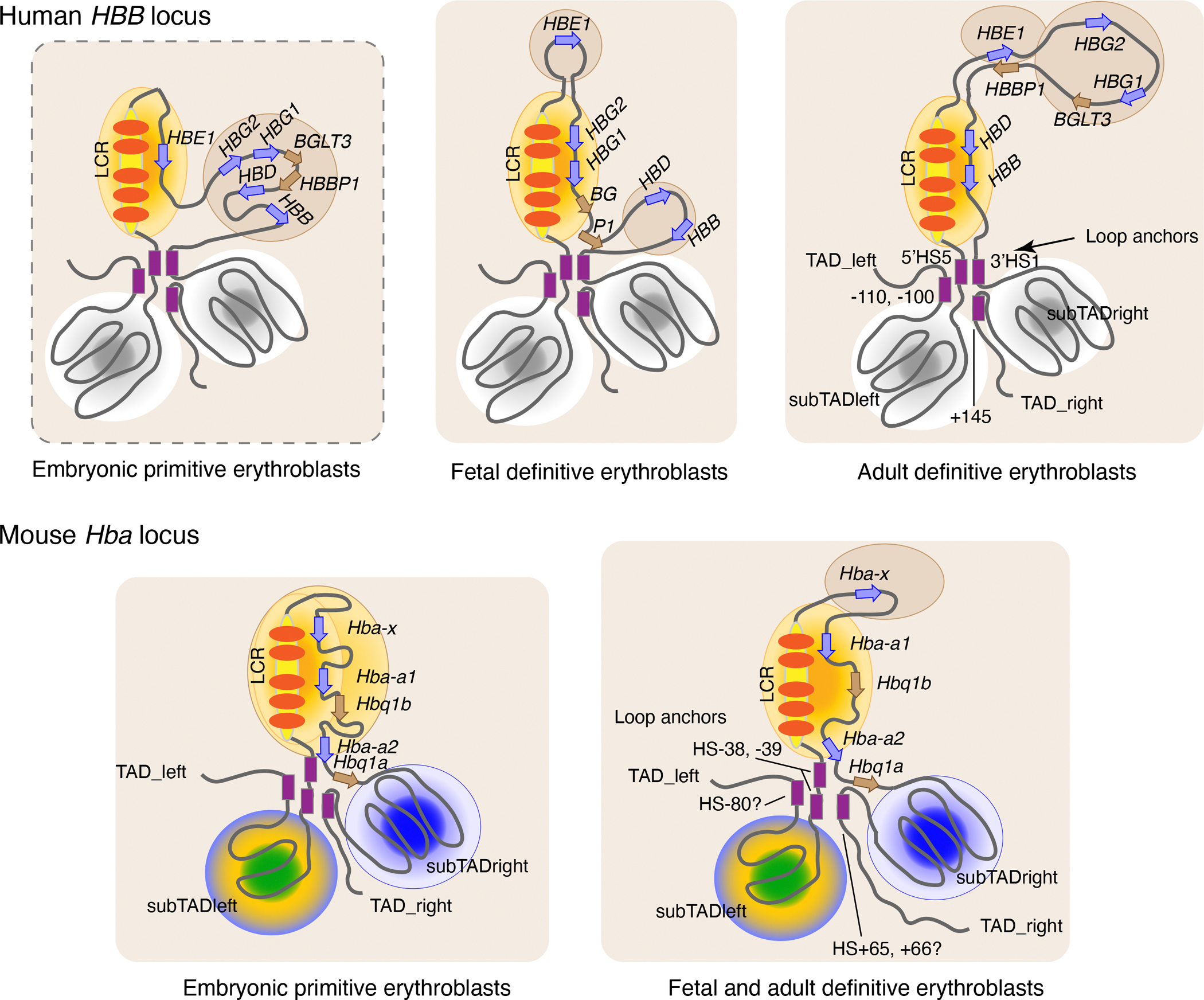
Differential expression of globin genes by changes in interactions with the LCR. In these conceptual illustrations, interaction with a transcriptionally active zone (hub, factory, biomolecular condensate, portrayed as a yellow-orange oval) anchored on the LCR leads to expression of a globin gene. Shifts in the genes interacting with the LCR active zone in erythroid cells at progressive stages of development result in differential expression. Shades of brown indicate repression. These different interactions are shown as occurring with the subTAD containing the LCR and globin genes. Similar shifts in patterns of interaction of genes with the LCR are indicated for both the *HBB* and *HBA* gene clusters. No information is currently available LCR-gene interactions for the *HBB* locus in embryonic primitive erythroblasts, indicated by the dashed outline around the illustration. Abbreviations: *BG* = *BGLT3*, *P1* = *HBBP1*.

Many lines of evidence indicate that the expression of individual alpha- or beta-like globin genes is regulated by contacts with their respective LCR (7; 68; 134; 144). These models have at least two themes in common: (a) elements within the LCR are close to each other in three dimensional space and in some way work together, and (b) differential expression of the genes within the cluster involves changes in the interaction frequency of the genes with the LCR. These concepts apply to both types of globin gene clusters, as illustrated in Figure 4 for the human beta-globin and mouse alpha-globin gene clusters.

Several results and prominent ideas are illustrated in Figure 4. The large excess of co-repressors (53) indicates that the overall nuclear milieu is repressive (light brown background). In contrast, TFs bound to HSs (red ovals) within the LCRs (long yellow ovals) recruit co-activators, remodelers, histone modifying enzymes, and the transcriptional machinery to generate a zone of high transcriptional activity (orange-red gradient oval). This zone may occupy substantial portions of the subTADs containing the globin gene clusters in erythroid cells, as suggested by the dominance of chromatin states indicative of activation throughout these subTADs (Figure 2). The high activity zone could be described as a transcription factory (119) or an active chromatin hub (114), or RNA POL2 clusters (30). At progressive stages of development, the expressed genes join the hub and are transcribed at high levels. Unexpressed genes are excluded from the hub, and their chromatin is repressed (brown background). Shifts in the interaction sites for a specific region, such as the *BGLT3* - *HBBP1* region in the beta-like gene cluster, can regulate LCR interactions (68). The activities of co-activators and histone modifying enzymes in the hub generate broad zones of histone acetylation, mediator binding, and BRD4 occupancy through the LCR and the target genes (Figure 3). These epigenetic features are general signatures of super enhancers. The hub may not have a specific macromolecular structure, but rather it could be a nucleation site for very high concentrations of proteins involved in gene activation, which come together though a large number of weak interactions, possibly forming a molecular condensate that serves as a membrane-less compartment within the nucleus (125). Further study, such as with super resolution microscopy (30), is needed to ascertain whether the zone of transcriptional activity has some structure, if it is an amorphous phase rich in activating proteins, or if it perhaps entails some combination of those models.

The general concept of a highly transcriptionally active zone anchored on the multi-component LCR that interacts selectively with target genes at progressive developmental stages provides a framework for synthesizing current data and identifying issues that need to be addressed (see Future Issues).

## 8. REGULATION OF HUMAN FETAL GLOBIN GENES AND APPROACHES FOR THEIR REACTIVATION

**Figure 5:**
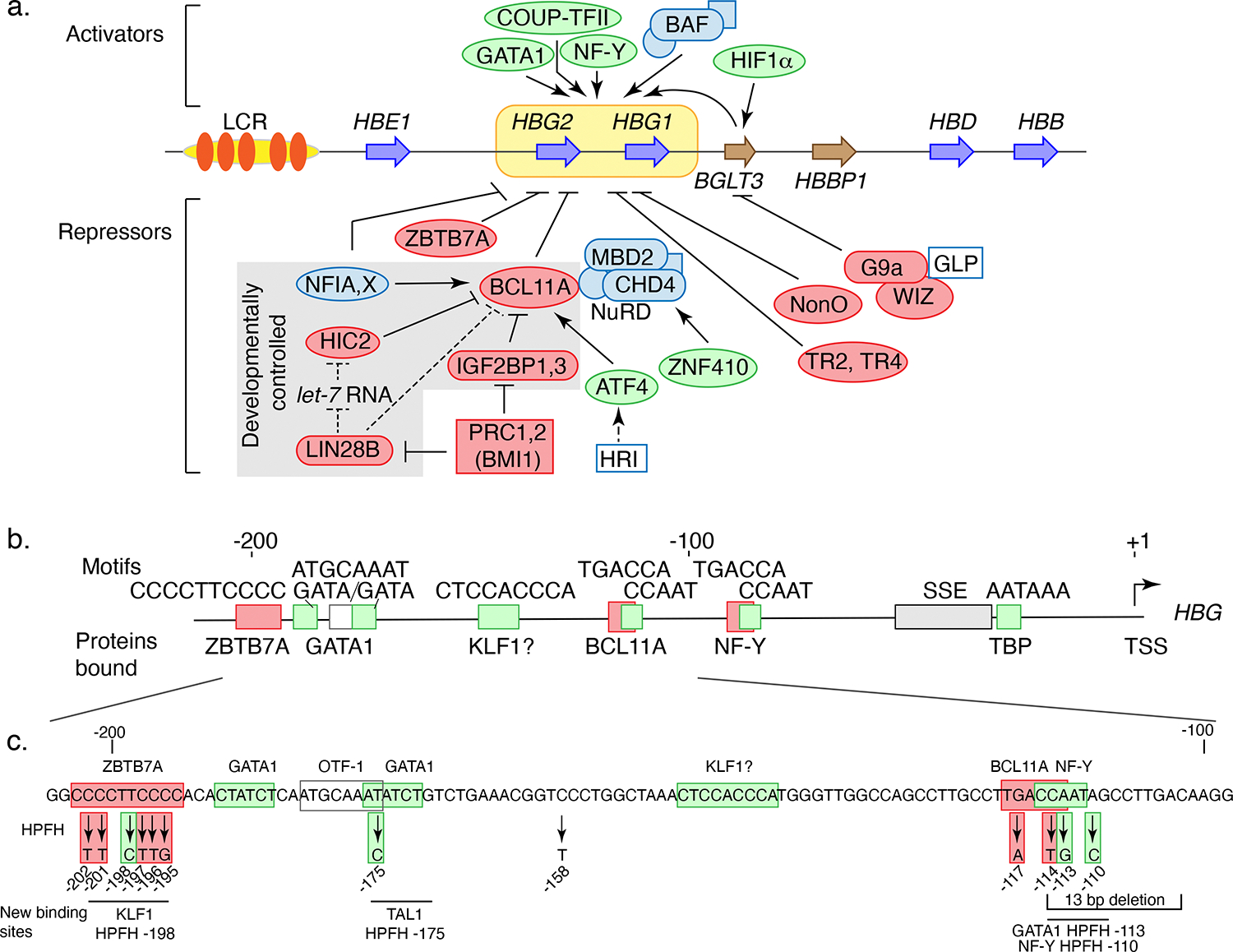
Proteins and elements regulating the proximal promoter of the *HBG1,2* genes. (*a*) Proteins and pathways regulating the developmental timing of expression of the *HBG1,2* genes. The lines for repression action are solid for direct effects and dashed for indirect effects. The ovals for proteins are shaded green for activators, red for repressors, and blue for complexes. (*b*) Regulatory elements and proteins implicated in regulation of the *HBG1,2* promoters. Protein binding sites motifs are shown as boxes along the line for the promoter, shaded in green for binding sites for activators and red for repressors. SSE is a stage selector element. (*c*) Alternations to the DNA sequence of the *HBG1,2* proximal promoters leading to HPFH. Along the DNA sequence, binding site motifs are boxed, proteins implicated in acting at those sites are above the sequence, and genetic changes leading to HPFH phenotypes are shown below the sequence. These are shaded in red for those the result in a decreased binding of the repressors and green for those that generate new binding sites for TFs. The proteins bound as a result of the latter substitutions are indicated at the bottom; the horizontal line covers the binding site motif generated by the relevant HPFH substitution. Panel *a* adapted and redrawn from Reference (78) with permission from Elsevier and Reference (44) (CC BY) with permission from the authors.

The quest to reactivate expression of fetal *HBG1,2* genes in adult erythroid cells has generated a rich trove of information on their regulation. Several rare genetic variants in the proximal promoters of the *HBG1,2* genes lead to high levels of HbF in adult erythroid cells in an inherited condition known as hereditary persistence of fetal hemoglobin (HPFH). Genome-wide association studies (GWAS) found common genetic variants associated with high HbF in and around genes not linked to the *HBB* gene cluster, including *BCL11A* (87; 102). Intensive study of these genetic variants and sensitive screens of CRISPR-Cas9 edited cells to find those expressing high levels of HbF have led to the discovery of many TFs and their sites of action in the promoters of *HBG1,2* genes (Figure 5).

Studies of proximal promoters of *HBG1,2* genes over several decades have implicated many TFs, including NF-Y (97), GATA1 (37; 96), and possibly KLF1 (5) (Figure 5a), as well as DNA methylation in their regulation (150). The discoveries of three major repressors of the *HBG1,2* genes, BCL11A, ZBTB7A, and the nuclear factor I family members NFIA and NFIX, have consolidated much previous information and improved our understanding of how HPFH variants lead to production of HbF in adult cells.

GWASs implicated variants in the *BCL11A* gene as contributing to elevated HbF (87, 102), and further detailed studies revealed that BCL11A is a major repressor of the *HBG1,2* genes (127; 166). While this TF is present broadly in many cell types and organs (92), the genetic variants affecting adult HbF production mapped to an erythroid-specific enhancer in *BCL11A* intron 2. Directed genome editing of key sites in that enhancer reduced BCL11A production in adult erythroid cells and led to increased expression of the *HBG1,2* genes (14). ChIP-seq and CUT&RUN mapping in adult erythroblasts revealed that BCL11A binds to the proximal promoter of the *HBG1,2* genes as well as to the LCR (90; 99) (Figure 3d). The binding site motif for BCL11A, TGACCA, which overlaps a CCAAT box, is duplicated in the *HBG1,2* genes, but BCL11A binds primarily to the distal site (relative to the transcription start site, or TSS) (91) (Figure 5b). This BCL11A binding site around position −115 is eliminated by several nucleotide substitutions and a short deletion that cause HPFH (Figure 5c).

A second major repressor of the *HBG1,2* genes is ZBTB7A (also called LRF). Directed genetic knockouts of the *ZBTB7A* gene caused a specific, large induction of the *HBG1,2* genes in adult erythroid cells independently of repression by BCL11A (101). ChIP-seq mapping showed that ZBTB7A binds to a site around position −200 in the proximal promoters of *HBG1,2* genes, which encompasses a second cluster of nucleotide substitutions that cause HPFH (99) (Figure 5b and c). While ZBTB7A, like BCL11A, has been implicated in roles in multiple cell types (95), it appears to have a specific role in repression of the *HBG1,2* genes in adult erythroid cells.

The TFs BCL11A and ZBTB7A work with the nucleosome remodeling and deacetylase co-repressor complex NuRD to down-regulate the *HBG1,2* genes in adult erythroid cells. Both TFs recruit NuRD to the *HBG1,2* promoters, where it deacetylates histones and establishes a repressive chromatin environment (101) (Figure 5a). The methyl-DNA reader protein MBD2 may facilitate this binding and maintain a repressive nucleosome over the basal promoter (131). This repressive environment can interfere with or block binding of transcriptional activators. For example, binding of BCL11A and NuRD to the distal TGACCA motif site blocks binding of the activator NF-Y to its nearby, proximal CCAAT binding site (37; 91).

The genetic alterations in the *HBG1,2* promoters that cause HPFH fall into two categories – loss of function variants that impair binding of a repressor and gain of function variants that create new binding sites for activators (Figure 5c). Most of the HPFH substitutions and deletions reduce the affinity of the repressors for their binding sites (99), thereby interfering recruitment of the co-repressor NuRD. By contrast, other HPFH substitutions generate new binding sites for TFs including TAL1, KLF1, GATA1, and NF-Y (37; 98; 160; 161) (Figure 5c). These gain of function substitutions lead to expression of the *HBG1,2* genes despite the presence of the repressors BCL11A and ZBTB7A. This ability of new binding sites to override repression shows that the repressive activities of BCL11A and ZBTB7A are not absolute (78; 161); this conclusion is supported by forced looping experiments in which juxtaposing the LCR with the *HBG1,2* genes also overcomes repression (36). Thus, it appears that additional factors could be involved in regulating the *HBG1,2* genes.

The repressor BCL11A is regulated by a remarkable variety of pathways (78) (Figure 5a). Several proteins previously not known to be involved in repression of the *HBG1,2* genes have been identified by CRISPR-cas9 screens for genes whose knock-down leads to higher expression of *HBG1,2* genes in HUDEP-2 cells, which normally express the *HBB* gene. The screens often employ guide RNAs specific for hundreds of genes encoding members of particular protein families to direct genome editing by CRISPR-cas9 in HUDEP2 cells, followed by validation in human primary erythroid cells (55; 136). These studies have revealed several pathways that regulate the production of BCL11A. For example, starting with a screen directed against genes encoding kinases (55), a series of experiments revealed a pathway in which heme-regulated inhibitor (HRI) catalyzes phosphorylation of the translation initiation factor eIF2alpha to increase the translation of mRNA for the TF ATF4 (69), which binds to the erythroid specific, intronic enhancer of *BCL11A* to boost its expression, thereby repressing the *HBG1,2* genes *(*Figure 5a). Other screens implicated the TF ZNF410 in regulating *HBG1,2* genes, showing that it binds with remarkable specificity to the *CHD4* gene to activate it and increase production of the CHD4 component of the NuRD complex, which is recruited by BCL11A for repression of the *HBG1,2* genes (85; 152). Analyzing screen results for hits that reduce expression of *HBG1,2* genes in HUDEP2 cells revealed HIC2 as a fetal-specific repressor of *BCL11A* (70). Translation of HIC2 in adult cells is inhibited by the microRNA *let-7* (71), which is inhibited by the RNA binding protein LIN28 in fetal cells. Under hypoxic conditions, the TF HIF1alpha can activate the *HBG1,2* genes, which reveals a role for this oxygen-sensing pathway in regulation of gamma-globin genes (42). Many additional proteins, including NFIA, X (121), and pathways are involved in regulation of the *HBG1,2* genes (Figure 5a), as described in recent reviews (44; 78).

It is not clear why so many diverse proteins and pathways regulate the *HBG1,2* genes. One possibility is that multiple, redundant repressive pathways could give robustness to the down-regulation in adult erythroid cells. No one pathway of repression causes an absolute silencing of the *HBG1,2* genes, but rather many different pathways lead to partial repression. The success of the directed screening approaches shows that small mutations can impair repression sufficiently to obtain a significant signal in the screens. This impact of small perturbations suggests that repression of the *HBG1,2* genes is not as severe as the silencing at loci that are refractory to activation, such as the *HBZ* gene in primitive erythroid cells (7; 80). This inferred relatively light repression of the *HBG1,2* genes in adult erythroid cells may be an important factor in current and developing therapeutic approaches, indicating that reactivation of the *HBG1,2* genes may be accomplished by small edits in the genome or by modulation of enzyme activity of the regulators using pharmacologically acceptable concentrations of inhibitors or agonists. Some of these possibilities are being realized with the approval of a genome editing strategy for treatment of sickle cell disease and beta-thalassemia (3; 48).

## 9. CONCLUDING REMARKS

Despite the differences in genomic context and evolutionary history of the alpha- and beta-globin gene clusters, their expression patterns are similar and balanced. This review summarizes many of the common features shared over multiple layers of regulatory control, including the arrangement of regulatory elements and genes, the topological constraint into a subTAD, the powerful impact of the multi-component LCRs located at the 5’ end of the gene clusters, the different activities of HS elements within the LCRs, including classical enhancers and facilitators, the common set of lineage-specific and widely distributed TFs and co-activators that act at the regulatory elements, and the apparent role of changes in contacts with the LCR in differential expression during development. It is likely that many aspects of these common regulatory features will apply more broadly to the regulation of many genes in vertebrates. However, the two types of gene clusters differ substantially in their genomic sequences, in the detailed patterns of expression during development, in the activity (or not) of surrounding genes in erythroid cells, and in other aspects. Indeed, the similarities emphasized in this review are not apparent in alignments of genomic DNA sequences, since almost no non-repetitive DNA segments align between the two clusters. By contrast, comparisons of epigenetic landscapes may reveal similarities with greater sensitivity (83; 164), and further pursuit of such approaches may be productive.

A major motivation for the study of globin genes has been to discover mutations, proteins, and regulatory pathways that impact the role of hemoglobins in disease, following the strategy that such mechanistic information would lead to the development of more effective therapies. This strategy is proving to be successful, with treatments based on the replacement of aberrant beta-globin via gene therapy and genome editing to reactivate fetal hemoglobin now being used in clinical practice. These advances highlight the success of basic research on gene structure and the fundamental mechanisms of gene regulation, but the current therapeutic approaches are expensive, resource-intensive, and not readily available to the world-wide population of patients (33). Ongoing research directions on additional or alternative approaches, such as using more specific genome editing approaches or developing pharmacological agents that modulate the pathways regulating *HBG1,2* expression, offer exciting challenges and potential hope for broadly applicable therapeutic advances.

## Supplementary Material

Hardison_GlbGeneReg_SupplTable

## FUTURE ISSUES

1. The signals and molecular mechanisms that move each globin gene into the active hub around the LCR at the appropriate stage are not fully known. Loops between the LCR and a target beta-globin genes can be formed by LDB1, but how it and other looping factors act in differential regulation is not yet known.
2. Do the components of the LCR or hub all come together in a relatively stable complex, or do the components act sequentially or in some other fashion? Super-resolution microscopy on individual chromosomes in living cells could provide answers.
3. The catalogs of candidate CREs predicted from epigenomic data for many cell types are extensive and possibly close to comprehensive. Assigning these to functional categories, such as promoters, classical enhancers, facilitators, insulators, and boundary elements, is an important task for the future.
4. Relationships among CREs need to be determined, e.g., which CREs work together, as in a super enhancer, and what are the gene targets for individual or groups of CREs?
5. More quantitative modeling is needed, such as determining the contribution of each CRE to the expression level of their target genes and elucidating the epigenetic features at each CRE to determine their contributions to expression levels.
6. What molecules and structures determine the boundaries of the TADs and subTADs?
7. Defining the structures and mechanisms that enable the TFs to find appropriate regulatory elements in DNA in chromatin remains a major challenge. Which TFs act as pioneer factors, with a demonstrable activity of opening chromatin by moving or displacing nucleosomes, and what signals lead to that activity?
8. A pseudogene, *HBBP1*, has been implicated in shifting chromatin interaction frequencies. Are other pseudogenes or genes with unknown functions involved in some structural role, possibly with an impact on chromatin interactions?
